# Supplementary material for: Case Report: Rare IKZF1 Gene Fusions Identified in Neonate with Congenital KMT2A-Rearranged Acute Lymphoblastic Leukemia
Source: Genes (Basel). 2023 Jan 19;14(2):264. doi: 10.3390/genes14020264 (PMC9956107; doi:10.3390/genes14020264)
Supplement: Supplementary file 1 [file genes-14-00264-s001.zip › genes-2128345-supplementary.pdf]

Table S1: Key characteristics of the B-ALL patient cohort used in the gene expression analyses. The cohort is comprised of 592 B-ALL patients, including the 4 samples from CHI_0391. Characteristics of the 588 additional samples are provided in the table. Note that *KMT2A::AFF1* and/or the reciprocal *AFF1::KMT2A* were the most common fusions observed (18 patients).

Disease timepoint	
Diagnosis	468
Relapse	119
Refractory	1
Age when diagnosed (years)	
Infant (≤1)	15
Child (1-15)	154
Adolescent (16-39)	158
Adult (≥40)	162
Unknown	99
Sex – no. (%)	
Male	289 (49)
Female	193 (33)
Unknown	106
Patients with gene fusions involving	
KMT2A	23
IKZF1	4
KDM2A	0
TUT1	0

Table S2: Complete range of gene fusions identified in patient CHI_0391 by transcriptomic sequencing

Gene 1	Gene 2	Fusion Description	Predicted Effect	Breakpoint 1	Breakpoint 2	Read Coverage 1	Read Coverage 2
Germline							
No clinically relevant gene fusions detected							
Diagnosis							
IKZF1	*TUT1*	oncogene, exon-exon	out-of-frame	chr7:50367353 (ex)	chr11:62349173 (ex)	918	703
KDM2A	*IKZF1*	oncogene, exon-exon	out-of-frame	chr11:66999431 (ex)	chr7:50444231 (ex)	120	712
KDM2A	*IKZF1*	oncogene, exon-exon	out-of-frame	chr11:66999431 (ex)	chr7:50435704 (in)	120	556
KDM2A	*IKZF1*	oncogene, exon-exon	out-of-frame	chr11:67007840 (in)	chr7:50435704 (in)	201	556
KDM2A	*IKZF1*	oncogene, exon-exon	out-of-frame	chr11:67007840 (in)	chr7:50444231 (ex)	201	712
KDM2A	*IKZF1*	oncogene, exon-exon	out-of-frame	chr11:66999431 (ex)	chr7:50450238 (ex)	120	234
KMT2A	*AFF1*	oncogene, exon-exon	in-frame	chr11:118355029 (ex)	chr4:88005272 (ex)	18	132
Refractory, Post-induction therapy							
IKZF1	*TUT1*	oncogene, exon-exon	out-of-frame	chr7:50367353 (ex)	chr11:62349173 (ex)	417	32
KDM2A	*IKZF1*	oncogene, exon-exon	out-of-frame	chr11:67007840 (in)	chr7:50444231 (ex)	21	218
KDM2A	*IKZF1*	oncogene, exon-exon	out-of-frame	chr11:67009646 (in)	chr7:50426012 (in)	10	43
KMT2A	*AFF1*	oncogene, exon-exon	in-frame	chr11:118355029 (ex)	chr4:88005272 (ex)	31	166
KMT2A	*AFF1*	oncogene, exon-exon	in-frame	chr11:118355029 (ex)	chr4:88005275 (ex)	31	166
On blinatumomab therapy							
IKZF1	*TUT1*	oncogene, exon-exon	out-of-frame	chr7:50367353 (ex)	chr11:62349173 (ex)	481	28

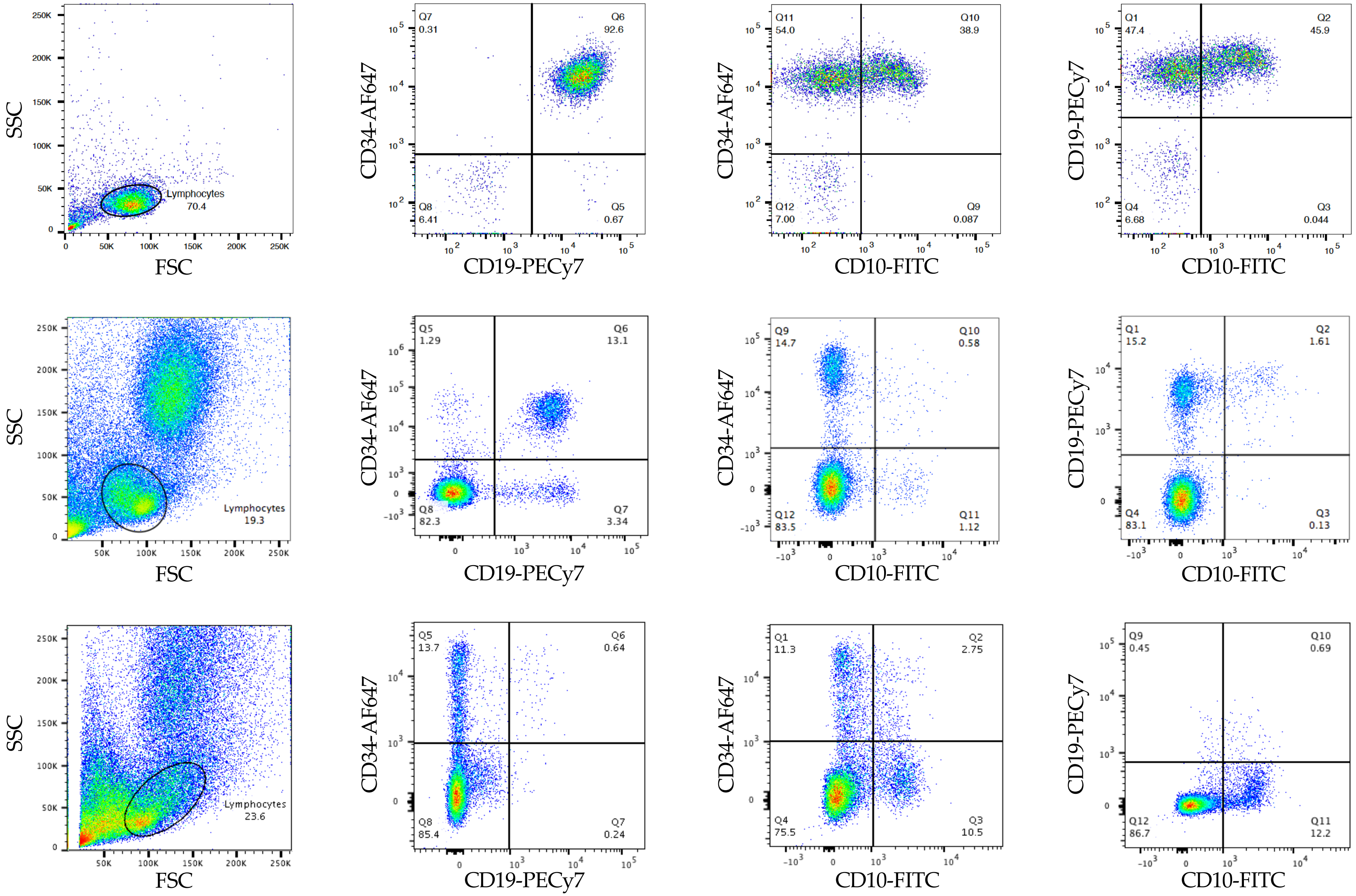

Figure S1. Immunophenotyping analyses of diagnosis (top), refractory (middle) and on-blinatumomab (bottom) samples demonstrated multiple populations of leukaemic blasts with differing CD10 and CD19 expression. The left panel shows the lymphoblast population upon which immunophenotyping was performed; the other panels show CD19, CD10 and CD34 expression.

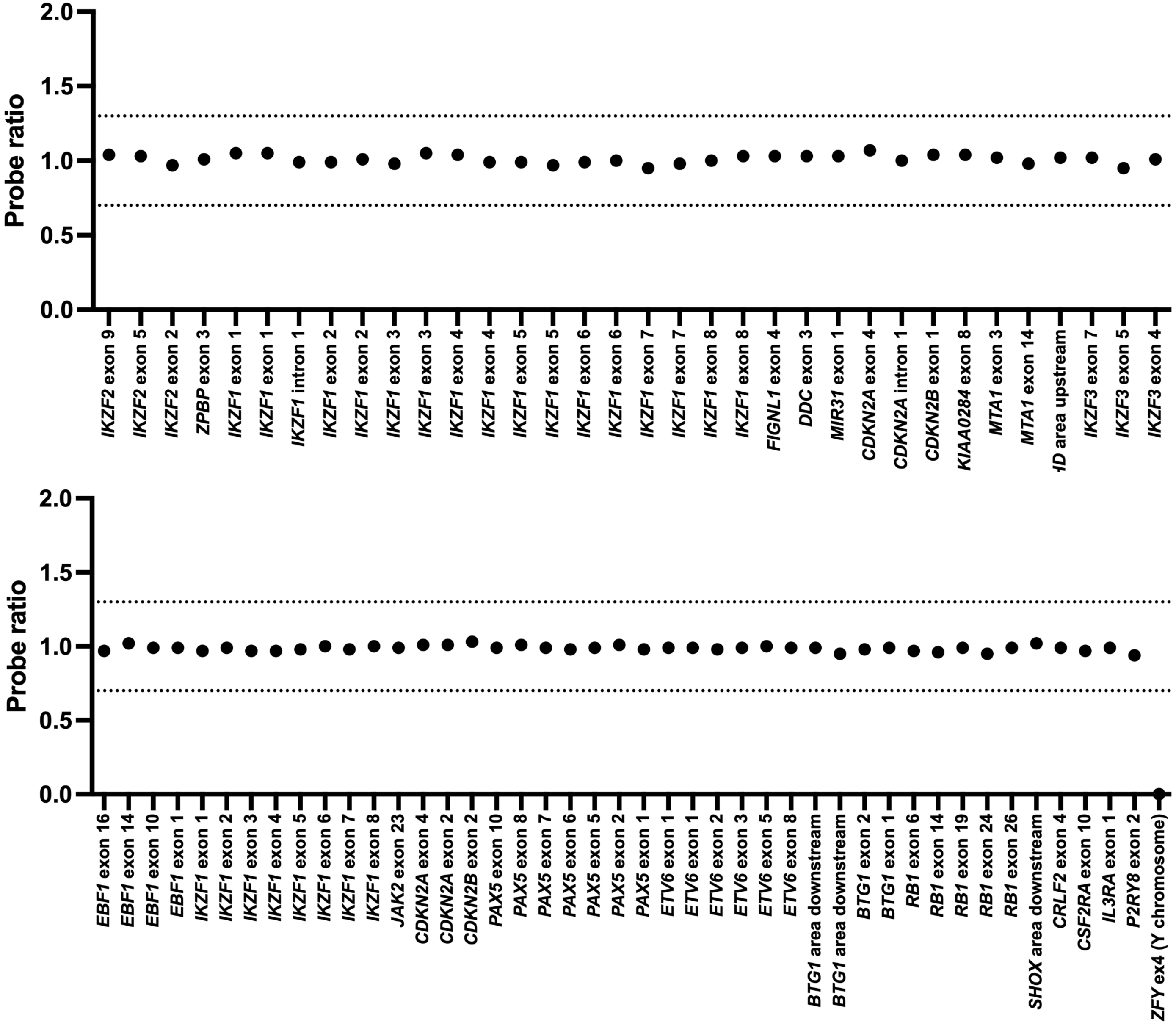

Figure S2. Analysis of deletions in key B-ALL genes in CHI_0391 were identified by multiplex ligand-dependent probe amplification (MLPA) on BMMNC using probes sets P202 (top) and P335 (bottom). No deletions or duplications (black dots) were identified in any of the genes included on these probe sets. Horizontal dotted lines represent threshold ratios for duplication and deletion. CPM=counts per million.
